# Adopting common data elements (CDEs) for the National Trauma Research Repository (NTRR): the results of an epidemiology Delphi survey

**DOI:** 10.1136/tsaco-2025-002095

**Published:** 2026-07-02

**Authors:** Jennifer Margaret Gurney, Nicolas W Medrano, Jeffrey A Bailey, Smith F Heavner, John B Holcomb, Molly P Jarman, Colleen M Ryan, Carl I Schulman, Jonathan Daniel Stallings, David T Harrington, Olga Volk

**Affiliations:** 1 Defense Committees on Trauma, Joint Trauma System, JBSA Fort Sam Houston, Texas, USA; 2 Department of Surgery, San Antonio Military Health System, San Antonio, Texas, USA; 3 Coalition for National Trauma Research, San Antonio, Texas, USA; 4 Surgery, Washington University in St Louis, St Louis, Missouri, USA; 5 Surgrey, Uniformed Services University of the Health Sciences, Bethesda, Maryland, USA; 6 Data Collaboration Center, Critical Path Institute, Tucson, Arizona, USA; 7 Discovery, Society of Critical Care Medicine, Mount Prospect, Illinois, USA; 8 Surgery, University of Alabama at Birmingham Health System, Birmingham, Alabama, USA; 9 Center for Surgery and Public Health, Brigham and Women’s Hospital, Boston, Massachusetts, USA; 10 Surgery, Mass General Brigham, Boston, Massachusetts, USA; 11 University of Miami School of Medicine, Miami, Florida, USA; 12 Data Enterprise, Joint Trauma System, Joint Base San Antonio Fort Sam Houston, Texas, USA; 13 Surgery, Brown University Warren Alpert Medical School, Providence, Rhode Island, USA; 14 Publicis Sapient, Arlington, Virginia, USA

**Keywords:** epidemiology, Surveys And Questionnaires, Research

## Abstract

**Introduction:**

To support the development of the National Trauma Research Repository (NTRR), a multidisciplinary workgroup used a consensus-driven approach to review established epidemiologic data elements and recommend basic common data elements (CDEs) for inclusion in the NTRR data dictionary.

**Methods:**

A 10-member workgroup of military and civilian trauma researchers and data scientists located and reviewed databases, codebooks, data collection forms, and published articles for data elements relevant to trauma epidemiology. Identified data elements were reviewed in a three-round Delphi survey, and monthly meetings with the workgroup were conducted. Consensus during the Delphi survey was analyzed with an 80% agreement threshold.

**Results:**

13 sources were reviewed for epidemiology data elements. After the three survey rounds and workgroup discussions, 33 elements (80%) reached consensus for inclusion, 3 elements (7%) were excluded, and 5 elements (12%) did not reach consensus.

**Discussion:**

The Delphi process proved effective in achieving expert consensus on basic CDEs for trauma epidemiologic research. The resulting standardized basic CDEs will improve data harmonization and support consistent data collection in the NTRR. These CDEs represent an initial framework and serve as a foundational starting point for epidemiological data collection within the NTRR. As researchers use the NTRR, the list of CDEs will grow and evolve with the needs of the trauma research community. CDE standardization not only supports interoperable research but also enhances research quality, efficiency, and translation into clinical practice.

**Level of Evidence:**

Level VII

**WHAT IS ALREADY KNOWN ON THIS TOPIC**

- Trauma epidemiology research is limited by inconsistent variable definitions and a lack of standardized, study-ready common data elements (CDEs), which restricts data comparability and reuse across studies. Although trauma registries and National Institutes of Health CDE efforts exist, they do not fully address the needs of cross-study epidemiologic research or repository-based data sharing.

**WHAT THIS STUDY ADDS**

- This study establishes a standardized set of National Trauma Research Repository (NTRR) Epidemiology CDEs with clear definitions and permissible values. These CDEs provide a practical, reusable minimum dataset aligned with NTRR submission and cross-study harmonization needs. The result is a shared epidemiologic framework that did not previously exist for trauma research.

**HOW THIS STUDY MIGHT AFFECT RESEARCH, PRACTICE OR POLICY**

- Use of these NTRR CDEs can improve interoperability, enabling pooled analyses and more efficient secondary use of trauma epidemiology data. Investigators may reduce study start-up time and data harmonization burden by adopting a common framework. The CDEs also support funder and journal expectations for standardized, reusable data in trauma research.

## Introduction

 Trauma remains a leading cause of death and disability across all age groups in the USA, presenting a persistent public health challenge with significant societal and economic impacts.^1^ Despite substantial progress in trauma care and prevention, trauma research remains hindered by a lack of standardized data elements that enable meaningful comparison and aggregation of findings across studies and institutions.^2^ The generalizability and reproducibility of trauma research, particularly within the trauma epidemiology field, are limited by the variability in how data are defined, collected, and reported.

As a key strategy for promoting data harmonization across research studies, common data elements (CDEs) are standardized, precisely defined variables intended to be consistently collected and used across studies within a given field. These CDEs facilitate data sharing, reduce duplication of effort, and enhance the ability to conduct large-scale analyses and meta-analyses.^3^,^5^ The adoption of CDEs can enable investigators to build more comprehensive, interoperable datasets that better support scientific discovery, policy development, and improvements in patient care, because improved efficiency in trauma research fosters the development of evidence-based guidelines.^6^ The Delphi method is a well-established, systematic approach for achieving consensus among a panel of experts, making it particularly suitable for selecting CDEs.^7^,^10^ This iterative process allows for anonymous input, controlled feedback, and statistical aggregation of group responses, all of which help to mitigate the influence of dominant individuals and support balanced decision-making.

The National Trauma Research Repository (NTRR) was established to serve as a centralized platform for storing, sharing, and curating trauma research data. As part of its development, the NTRR convened a series of expert workgroups to identify CDEs in specific study environments or phases of care. These included epidemiology, prehospital care, acute hospital care, rehabilitation, and social determinants of health. The process was iterative and allowed anonymous input, which helped moderate the influence of dominant individuals.

The Epidemiology Workgroup’s goal was to identify and prioritize CDEs relevant to trauma epidemiology. Epidemiological data from both civilian and military trauma populations is essential to collect in trauma studies, as it enables a comprehensive understanding of the incidence, distribution, outcomes, and determinants of injury patterns across different populations and settings.^2 11^ This data is also crucial for identifying risk factors, informing prevention strategies, optimizing trauma care systems, and guiding resource allocation.^12^ Epidemiological data enables system-level analysis and supports policy decisions. Epidemiologic data are crucial elements in trauma research from both an overall outcomes perspective and to influence prevention. Standardization in this domain ensures that trauma care can be guided by population-level evidence. Integrating this process with military trauma data standards also allows for interoperability and supports the development of military–civilian best practices in trauma care. By capturing epidemiologic variables such as injury mechanism, injury severity, and demographics, researchers can track trends, evaluate interventions, and improve clinical protocols. Furthermore, epidemiological surveillance facilitates international comparisons, ultimately contributing to reductions in injury-related morbidity and mortality.

This article describes the systematic approach undertaken by the NTRR Epidemiology Workgroup to identify common epidemiological data elements, defined as data elements recommended to be collected by trauma studies and suitable for inclusion in the NTRR. Using a structured Delphi consensus process, the workgroup engaged a diverse panel of subject matter experts in trauma epidemiology to review, refine, and prioritize candidate CDEs. The goal of this process was to develop a consensus-driven set of basic CDEs that would enhance the quality, consistency, and utility of epidemiological trauma research conducted using the NTRR.

## Methods

Members of the NTRR Executive Steering Committee and the NTRR CDE Steering Committee recommended subject matter experts for the NTRR Epidemiology Workgroup and several other workgroups focused in different environments of care. Coalition for National Trauma Research (CNTR) gathered information on those recommended to serve, including work environment, trauma subspecialty, research experience, geographic location, and military experience, in an effort to comprise workgroups with diverse perspectives from across the USA. After guidance from National Institutes of Health regarding effective Delphi groups, we sought to form workgroups of roughly a dozen members each. A workgroup of 10 subject matter experts was convened to serve on the NTRR Epidemiology Workgroup. The workgroup was multidisciplinary, including trauma surgeons, burn specialists, trauma system organizers, epidemiologists, and data scientists, and included both civilian and military representatives. As this project did not involve human subjects, Institutional Review Board approval was not required.

To identify CDEs relevant to trauma epidemiology, workgroup members compiled relevant and applicable studies and existing databases for review. From these resources, we extracted individual data elements and conducted a frequency analysis to analyze which variables were most common across studies. Based on this analysis, we selected the top 50 most used data elements, including all elements tied in frequency with the 50th-ranked element (n=65). Certain data elements were then removed either because they had already been recommended for adoption as a core CDE or were deemed not applicable to the workgroup’s scope (online supplemental 1).^13^ Participant engagement, respondent burden, timeline, and project feasibility were considered when setting the parameters for the number of data elements to include. To ensure consistency and promote alignment with existing standards, definitions for each data element were gathered from established repositories. Definitions were selected after a predefined hierarchy of precedence.^13^

CNTR gained extensive experience in configuring, running, and analyzing Delphi surveys through its development of the National Trauma Research Action Plan. That project engaged more than 400 multidisciplinary stakeholders participating in 11 different Delphi panels to identify research gaps and high-priority research questions that should direct extramural research support in the future. Thus, we built on our familiarity with Calibrum’s Surveylet platform to design a similar approach to drive consensus on the basic CDEs to include in the NTRR data dictionary. Although the Delphi survey was initially designed to incorporate a traditional 5-point Likert scale, the NTRR Executive Steering Committee recommended an abbreviated three-response approach to reduce participant cognitive load and avoid regression to the mean.

We created a Delphi survey using Calibrum’s Surveylet application, populating it with the data elements previously identified along with their definitions and permissible values (PVs), after the order of precedence.^13^ The workgroup proceeded through three rounds of consideration in the online Delphi survey, where they rated whether an element should be included in the NTRR through the response options of “Include, “Exclude,” or “No opinion.” During round 1, workgroup reviewers had the opportunity to anonymously (1) rate whether an element should be included in the NTRR, (2) rank the currently existing definitions/PVs of the data element in order of preference for adoption (rank 1 being most preferred), (3) leave comments describing their rationale regarding the inclusion of a data element or preference of definition, and (4) recommend additional data elements for inclusion. For a data element to reach consensus for inclusion in the NTRR, an 80% agreement threshold was set. Definition selection was based on the definition receiving the lowest mean ranking (ie, closest to 1). Data elements that reached consensus were hidden in subsequent rounds so that workgroup members considered only those elements that had not yet reached consensus.

The workgroup completed the three rounds of the Delphi from May to October 2024, during which time they received email reminders to encourage timely completion of each round. A total of eight (80%) workgroup members participated in round 1, six (60%) in round 2, and eight (80%) in round 3. The workgroup also met monthly via teleconference to discuss outstanding items for review, including (1) comments left by reviewers, (2) data elements with tied rankings on their definitions and PVs, (3) creation of definitions/PVs for data elements that did not exist within the repositories in the order of precedence, (4) data elements selected for inclusion by more than one NTRR workgroup, and (5) adoption of form structures to more effectively collect specific data element groupings. All responses from the Delphi were kept anonymous when shared with the group; however, members were identifiable when providing feedback during the teleconference meetings. Consensus decisions made during these approximately 1 hour meetings informed revisions to the Delphi survey and the final selection of CDEs for inclusion. On completion of these discussions, results were shared with the NTRR CDE Steering Committee and NTRR Executive Committee for final approval.

## Results

**Figure 1:**
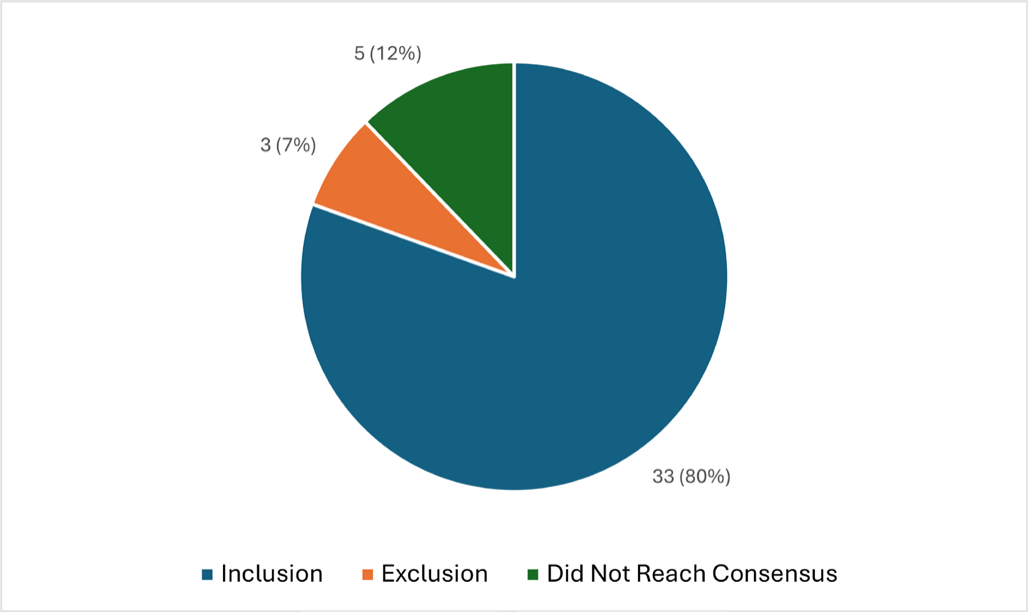
Data element results from Delphi and post-Delphi discussion.

**Figure 2:**
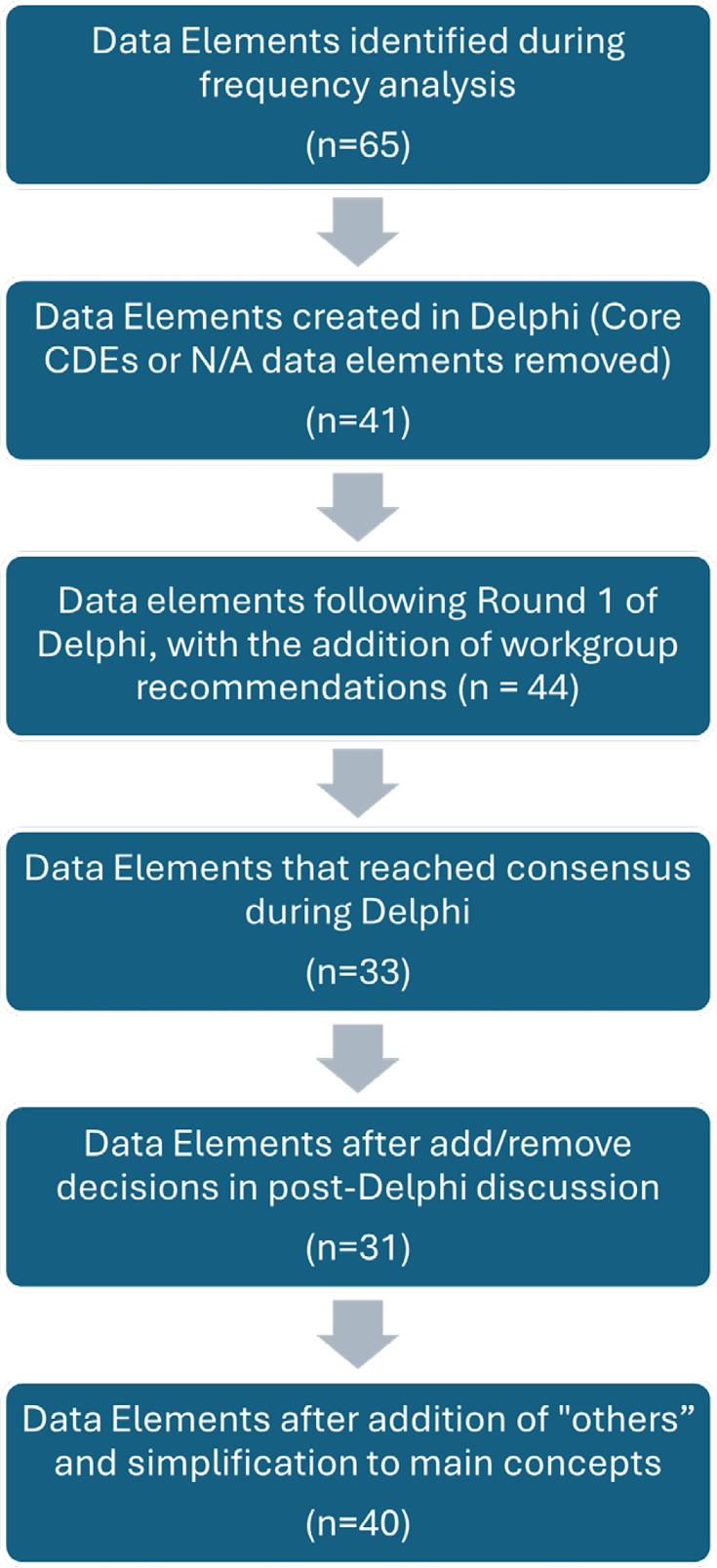
Number of data elements through CDE selection process. CDEs, common data elements; N/A, not applicable.

13 sources were reviewed for epidemiological data elements (box 1), from which 41 elements were included in the first round of the Delphi survey. After the first round and the addition of workgroup recommendations, there were 44 data elements in the Delphi. On completion of the three Delphi rounds and post-Delphi workgroup meetings, 33 (80%) data elements reached consensus for inclusion (box 2), 3 (7%) reached consensus for exclusion (online supplemental 2), and 5 (12%) did not reach consensus (figure 1S 3). After the addition of “Other” data elements to capture instances where “Other, specify” was a PV, and simplification of data elements to main concepts (box 2), 40 elements were recommended for adoption in the NTRR (figure 2S 4).

Box 1 **Sources reviewed for analysis of frequently collected data elements.**

- Federal Interagency Traumatic Brain Injury Research Informatics System (FITBIR)—Epidemiology Common Data Elements.**^14^**
- National Trauma Data Standard (NTDS).**^15^**
- National Highway Traffic Safety Administration—Fatality Analysis Reporting System (FARS).**^16^**
- Federal Bureau of Investigation Uniform Crime Reporting Program.**^17^**
- United States Coast Guard Marine Casualty Reporter.**^18^**
- Department of Defense Trauma Registry (DODTR).**^19^**
- American Burn Association—Burn Care Quality Program (BCQP).**^20^**
- Federal Aviation Administration—National Transportation Safety Board Aviation Accident and Incident Data System.**^21^**
- Armed Forces Medical Examiner Registry.**^22^**
- Multi-institutional Multi-disciplinary Injury Mortality Investigation in the Civilian Pre-Hospital Environment.**^12^**
- Saving Lives on the Battlefield: A Joint Trauma System Review of Pre-Hospital Trauma Care in Combined Joint Operating Area Afghanistan (CJOA-A) Executive Summary.**^23^**
- The Effect of a Golden Hour Policy on the Morbidity and Mortality of Combat Casualties.**^24^**
- Saving Lives on the Battlefield (Part II)—One Year Later: A Joint Theater Trauma System and Joint Trauma System Review of Prehospital Trauma Care in Combined Joint Operations Area Afghanistan (CJOA-A) Final Report.**^25^**

Box 2 **Data elements that reached consensus for inclusion in the NTRR.**

**Patient characteristics**

- Age.
- Age units.
- Patient’s Home State.†
- Patient’s Home City.†
- Patient’s Home Zip/Postal Code.†
- Patient’s Occupation.
- Patient’s Occupation Specify Other.*
- Marital Status.
- Highest Level Education.
- Employment Status.
- Employment Status Specify Other.*
- Insurance Status.
- Insurance Status Specify Other.*
- Medical history condition SNOMED CT Code.

**Injury characteristics**

- Protective Device Type.
- Protective Device Type Specify Other.*
- Responders Involved.
- Responders Involved Specify Other.*
- Description of Events Text.
- Injury/Incident Date/Time.
- ICD-10 Place of Occurrence External Cause Code/Injury Place Occurrence.
- ICD-10 Injury Diagnoses.
- Manner of Injury (Accident, Homicide, etc.).
- Trauma Type (Blunt, penetrating, etc.).
- Trauma Type Specify Other.*
- Manner (cause) of Death.
- Pre-existing Conditions.
- Pre-existing Conditions Specify Other.*
- Vital Status.
- Incident Country.
- Incident State.†
- Incident City.†
- Incident Location Zip/Postal Code.†
- Military deployment injury indicator.
- Injury Severity Score (ISS).

**Facility**

- Inter-Facility Transfer Indicator.
- Alcohol Screen Indicator.
- Alcohol Screen Results.
- Drug Screen Indicator.
- Drug Screen Results.
- Drug Screen Results Specify Other.*

*Elements were not included in the delphi, but were created due to “Other, specify” permissible values.†Elements reached consensus but were simplified to main concepts—see discussion.NTRR, National Trauma Research Repository.

For data elements that did not reach consensus for inclusion or that met consensus for exclusion, three primary themes emerged. These elements were most often considered too narrowly focused to be broadly applicable across studies, offered limited added value, or were judged redundant because similar elements already captured the relevant information. For example, the data elements Education Participation and Patient Occupation Industry were not added because their information was already encompassed by the adopted elements Highest Level of Education and Patient Occupation, respectively.

## Discussion

The systematic approach used by the workgroup successfully identified a set of basic CDEs to support trauma epidemiology research within the NTRR. Through a rigorous review of existing resources and a structured Delphi consensus process, we were able to build consensus on most of the candidate data elements. The high rate of inclusion (83%) suggests strong alignment among participating experts regarding fundamental variables necessary for trauma epidemiology studies. The 15% of elements that did not reach consensus likely highlight areas where further clarification or development may be needed, either due to differences in current practice, lack of standard definitions, or evolving research priorities.

Many of the data elements that reached consensus during the Delphi survey were not identified as “main concept” CDEs in their original form, such as: Patient Home State, Patient Home City, Patient Home Zip Code, Incident State, Incident City, and Incident Zip Code. These data elements are seen as too context-specific. Since these elements fundamentally capture the same concept, regardless of the contextual label (eg, home, incident, treatment), they would lead to an unnecessary number of nearly identical elements. To reduce redundancy and limit the creation of additional state, city, and zip code variables, these elements were consolidated under existing Federal Interagency Traumatic Brain Injury Research Informatics System CDEs. Standard CDEs will be used within form structures to capture both “patient home” and “incident” location information. This approach ensures comprehensive location data collection without the need to create six new CDEs. The Delphi process of selecting CDEs is not without its limitations. The process reflects the perspectives of the participants involved, and therefore, the studies and projects reviewed for data elements were collected based on workgroup members’ knowledge and previous experience. The participants have historically been primarily affiliated with US trauma systems and therefore may introduce systemic bias toward data elements or definitions that are most familiar or routinely used within the US context. Thus, some essential studies may have been overlooked. Additionally, the decision to limit the Delphi survey to approximately 50 data elements, although practical, may have excluded less frequently used but still important variables. Finally, many of the sources included were projects conducted in the USA and, therefore, the recommendations provided may not apply to researchers and studies conducted outside of the USA.

The success of this effort reinforces the importance of using a Delphi process for developing CDEs. The iterative survey rounds, combined with structured expert discussion, allowed for refinement of both the data elements themselves and their associated definitions and PVs. Specifically, monthly workgroup meetings played a critical role in resolving instances where more than one data element for a specific topic reached consensus.

This effort represents a foundational step in improving trauma care through data-driven insights and data interoperability. By harmonizing critical epidemiological variables in both civilian and military research domains and standardizing how they are collected and defined, military and civilian researchers can build interoperable datasets that support large-scale, high-quality research across diverse settings. This level of standardization enhances the efficiency and interoperability of trauma research and ideally will accelerate the identification of life-saving interventions and enable benchmarking and continuous quality improvement within and across trauma systems. Moreover, epidemiological CDEs can serve as a bridge between individual patient encounters and system-level readiness, informing both public health prevention strategies and combat casualty care innovations. Including the military trauma system in this effort is an important step in aligning military and civilian trauma systems to capitalize on research investments and insights. Ideally, a unified and standardized approach to epidemiological data not only strengthens the scientific foundation of trauma research but also ensures that knowledge translates into improved survivability and better outcomes for all trauma patients—in the USA, on the battlefield and beyond.

The selection of epidemiological CDEs presented here represents an initial framework, intended to serve as a foundational starting point for data collection within the NTRR. As researchers design and complete studies and contribute their data to the NTRR, the list of CDEs is expected to evolve. New data elements may be proposed based on emerging research needs, novel methodologies, gaps identified through real-world use, and integration of international researchers. This iterative process ensures that the CDEs remain responsive to the needs of the trauma research community, supporting both standardization and innovation over time. Looking to the future, the NTRR aims to function as a centralized research infrastructure that facilitates standardized data harmonization and sharing, supports interdisciplinary collaboration, and advances the pace and impact of trauma research.

## Supplementary material

10.1136/tsaco-2025-002095 online supplemental file 1

## Data Availability

No data are available.
