# Supplementary material for: Adopting common data elements (CDEs) for the National Trauma Research Repository (NTRR): the results of an epidemiology Delphi survey
Source: Trauma Surg Acute Care Open. 2026 Jul 2;11(Suppl 3):e002095. doi: 10.1136/tsaco-2025-002095 (PMC13331208; doi:10.1136/tsaco-2025-002095)
Supplement: online supplemental file 1 [file tsaco-11-Suppl_3-s001.pdf]

Data Element	Frequency	Percent
Injury/Incident Date	12	92%
*Age	10	77%
*Race	9	69%
ICD-10 Place of Occurrence External Cause Code	9	69%
*ICD-10 Primary External Cause Code	9	69%
*Ethnicity	8	62%
Injury/Incident Time	8	62%
Date of Birth	8	62%
Initial ED/Hospital Weight	7	54%
Transport Mode	7	54%
First name	7	54%
Last name	7	54%
Vehicular protective device type	7	54%
*Gender	6	46%
Vehicular protective device other text	6	46%
Pre-Existing Conditions/Comorbidities	6	46%
ICD-10 Injury Diagnoses	6	46%
Incident Country	6	46%
Manner of Injury (Accident, Homicide, etc)	6	46%
Incident City	5	38%
*Sex	5	38%
Alcohol Screen	5	38%
Drug Screen	5	38%
Hospital Complications	5	38%
ICD-10 Hospital Procedures	5	38%
Initial ED/Hospital GCS-Eye	5	38%
Initial ED/Hospital GCS-Motor	5	38%
Initial ED/Hospital GCS-Total	5	38%
Initial ED/Hospital GCS-Verbal	5	38%
Initial ED/Hospital Temperature	5	38%
Inter-Facility Transfer	5	38%
Total ICU Length of Stay	5	38%

Data Element	Frequency	Percent
Total Ventilator Days	5	38%
Military ISS	5	38%
Trauma Type (Blunt, penetrating, thermal, etc)	5	38%
*AIS Code	5	38%
*AIS Body Region Category	5	38%
Responders Involved	5	38%
ED Discharge Disposition	5	38%
Patient's Home City	4	31%
Patient's Home State	4	31%
Patient's Home Zip/Postal Code	4	31%
Incident Location Zip/Postal Code	4	31%
Incident State	4	31%
Incident Latitude	4	31%
Incident Longitude	4	31%
24 Hour Total Blood Products	4	31%
24 Hour Total Colloid	4	31%
24 Hour Total Crystalloid Fluids	4	31%
Description of Complications Text	4	31%
ED Discharge Date	4	31%
ED Discharge Time	4	31%
ED/Hospital Arrival Date	4	31%
ED/Hospital Arrival Time	4	31%
Initial ED/Hospital Oxygen Saturation	4	31%
Initial ED/Hospital Pulse Rate	4	31%
Initial ED/Hospital Respiratory Rate	4	31%
Initial ED/Hospital Systolic Blood Pressure	4	31%
Military deployment injury indicator	4	31%
Max AIS Military	4	31%
Military New ISS	4	31%
Discharge Weight	4	31%
AIS Civilian Severity	4	31%
AIS Military Severity	4	31%
ISS	4	31%

Supplemental 1. Data element frequency from literature review.

Data Element
Education Participation
Date of Birth
Patient Occupation Industry

Supplemental 2. Data elements that reached consensus for exclusion from the NTRR.

Data Element
Traffic accident self alcohol influence likelihood type
Traffic accident self drug influence likelihood type
Incident Latitude
Incident Longitude
Weather Code

Supplemental 3. Data elements that did not reach consensus.

DATA ELEMENT	DEFINITION SOURCE	VARIABLE TITLE	DATA DEFINITION	PERMISSIBLE VALUES
Age	NTDS	Age	The patient's age at the time of injury (best approximation)	Numeric (Free-Form Entry)
Age units	NTDS	Age units	The units used to report the patient's age	Hours | Days | Months | Years | Minutes | Weeks
Address State Code	FITBIR	Address state code	The state code for the address (to describe where a mail piece is intended to be delivered).	Two-digit numeric FIPS code (Free-Form Entry)
Address state other text	FITBIR	Address state other text	A free-text field related to Address state code specifying other text	Alphanumeric (Free-Form Entry)
Address city name	FITBIR	Address city name	Name of the city or township for the address (to describe where a mail piece is intended to be delivered)	Alphanumeric (Free-Form Entry)
ZIP partial code	FITBIR	ZIP partial code	First three digits of the zip code where the participant/subject currently lives.	Numeric (Free-Form Entry)
ZIP code	Workgroup	ZIP code	First five digits of the Zip code	Numeric (Free-Form Entry)

DATA ELEMENT	DEFINITION SOURCE	VARIABLE TITLE	DATA DEFINITION	PERMISSIBLE VALUES
Patient's Occupation	FITBIR	Job classification pre-injury	Job classification pre-injury	Agricultural or fishery worker (vegetable grower, livestock producer, etc) | Armed forces (government military) | Clerk (secretary, cashier, etc) | Craft or trades worker (carpenter, painter, jewelry worker, butcher, etc) | Elementary worker (street food vendor, shoe cleaner, etc) | Legislator, senior official or manager | Not applicable (Not working pre-injury/not working now) | Other, specify | Plant/machine operator or assembler (equipment assembler, sewing-machine operator, driver, etc) | Professional (engineer, doctor, teacher, clergy, etc) | Service or sales worker (cook, travel guide, shop salesperson, etc) | Technician or Associate Professional (inspector, finance dealer, etc) | Unknown
Patient's Occupation Specify Other	Workgroup	Job classification pre-injury other	The free-text field related to "Patient's Occupation" specifying other text.	Alphanumeric (Free-Form Entry)
Marital Status	NIH	Marital Status	A demographic parameter indicating a person's current conjugal status	Married | Living as married or living with a romantic partner | Married or partnered, but not living together | Divorced | Widowed | Separated | Single, never been married-not living with romantic partner | Prefer not to answer
Highest level education	FITBIR	Highest level education pre-injury	Highest level education pre-injury	None/basic primary=0-4yrs | Post Graduate=18-19yrs (e.g., Masters, Doctoral) | Primary/Intermediate Primary=5-8yrs | Secondary=12yrs | Tertiary=13-15yrs (e.g., Teachers, Technicians) | University=16-17yrs | Unknown

DATA ELEMENT	DEFINITION SOURCE	VARIABLE TITLE	DATA DEFINITION	PERMISSIBLE VALUES
Employment Status	FITBIR	Employment status	Status of participant/subject's current employment	Disabled, permanently or temporarily | Keeping house | Looking for work, unemployed | Only temporarily laid off | Other, specify | Retired | Sick leave or maternity leave | Student | Unknown | Working now
Employment Status Specify Other	Workgroup	Employment Status Other	The free-text field related to "Employment Status" specifying other text	Alphanumeric (Free-Form Entry)
Insurance Status	NINDS	Health Insurance Type	Type of insurance that participant/subject currently has providing coverage for medical, surgical or hospital care	Medicaid | Medicare | Employer-sponsored disability insurance | Private or group health insurance | National Health Insurance | Veterans Affairs/Military | No insurance/self-pay | Unknown | Other, specify
Insurance Status Specify Other	Workgroup	Health Insurance Type Other	The free-text field related to "Insurance Status" specifying other text	Alphanumeric (Free-Form Entry)
Medical history condition SNOMED CT Code	FITBIR	Medical history condition SNOMEDCT code	Systematized Nomenclature Of Medicine Clinical Terms (SNOMED CT) code for medical condition/disease reported by the participant/subject	Numeric (Free-Form Entry)
Protective device type	NTDS	Protective Devices	Protective devices (safety equipment) in use or worn by the patient at the time of the injury	None | Lap belt | Personal floatation device | Protective Non-clothing gear (e.g. shin guard) | Eye protection | Child restraint | Helmet (e.g. bicycle, skiing, motorcycle) | Airbag present | Protective Clothing (e.g. padded leather pants) | Shoulder belt | Other, specify
Protective device type Specify Other	Workgroup	Protective device type other	The free-text field related to "Protective device type" specifying other text	Alphanumeric (Free-Form Entry)

DATA ELEMENT	DEFINITION SOURCE	VARIABLE TITLE	DATA DEFINITION	PERMISSIBLE VALUES
Responders Involved	FITBIR	Emergency service type	Branch of service involved at the accident scene	Ambulance (Core EMT-B) | Ambulance with specialized personnel (EMT-1) | Firefighter | Helicopter medical service | None | Other, specify | Police
Responders Involved Specify Other	Workgroup	Responders Involved Other	The free-text field related to "Responders Involved" specifying other text	Alphanumeric (Free-Form Entry)
Description of Events Text	FITBIR	Injury Description Text	A text description of the injury event	Alphanumeric (Free-Form Entry)
Injury/Incident Date Time	FITBIR	Injury date time	Date (and time, if applicable and known) of injury	Date or Date & Time ISO 8601 (Free-Form Entry)
ICD-10 Place of Occurrence External Cause Code/Injury place occurrence	NTDS	ICD-10 place of occurrence external cause code	Place of occurrence external cause code used to describe the place/site/location of the injury (Y92.X)	Numeric (Free-Form Entry)
ICD-10 Injury Diagnoses	NTDS	ICD-10 injury diagnosis	Diagnoses related to all identified injuries	Numeric (Free-Form Entry)

DATA ELEMENT	DEFINITION SOURCE	VARIABLE TITLE	DATA DEFINITION	PERMISSIBLE VALUES
MannerofInjury(Accident, Homicide, etc.)	FITBIR	Injury cause type	The type as related to the classification of the external cause of the injury	Accidental falls | Accidental poisoning by drugs, medicinal substances, and biologicals | Accidental poisoning by other solid and liquid substances, gases, and vapors | Accidents caused by fire and flames | Accidents caused by submersion, suffocation, and foreign bodies | Accidents due to natural and environmental factors | Air and space transport accidents | Drugs, medicinal and biological substances causing adverse effects in therapeutic use | Homicide and injury purposely inflicted by other persons | Injury resulting from operations of war | Injury undetermined whether accidentally or purposely inflicted | Late effects of accidental injury | Legal intervention | Misadventures to patients during surgical and medical care | Motor vehicle nontraffic accidents | Motor vehicle traffic accidents | Other accidents | Other road vehicle accidents | Railway accidents | Suicide and self-inflicted injury | Surgical and medical procedures as the cause of abnormal reaction of patient or later complication, without mention of misadventure at the time of procedure | Terrorism | Vehicle accidents not elsewhere classifiable | Water transport accidents
Injury Mechanism (Trauma Type)	Workgroup	Manner of Injury Type	Manner of injury type	Blast | Blunt | Burn | Penetrating | Other, specify
Injury Mechanism Specify Other	Workgroup	Mechanism of Injury Other	The free-text field related to "Injury Mechanism" specifying other text	Alphanumeric (Free-Form Entry)

DATA ELEMENT	DEFINITION SOURCE	VARIABLE TITLE	DATA DEFINITION	PERMISSIBLE VALUES
Manner (cause) of death	FITBIR	Death cause ICDD-CM code	ICD-CM code that describes the cause of Participant's/subject's death	Numeric (Free-Form Entry)
Pre-existing conditions	Workgroup	Pre-existing Condition	A condition/disease (occurring prior to injury) reported by the participant/subject or documented in the medical record as part of medical history	Other, specify | Alcohol Use Disorder | Bleeding Disorder | Currently Receiving Chemotherapy for Cancer | Congenital Anomalies | Congestive Heart Failure | Current Smoker | Chronic Renal Failure | Cerebrovascular Accident | Diabetes Mellitus | Disseminated Cancer | Advanced Directive Limiting Care | Functionally Dependent Health Status | History of Angina Within 30 days | History of Myocardial Infarction | History of Peripheral Vascular Disease | Hypertension | Prematurity | Chronic Obstructive Pulmonary Disease | Steroid Use | Cirrhosis | Dementia | Major Psychiatric Illness | Drug Use Disorder | Attention Deficit Hyperactivity Disorder | Anticoagulant Therapy | Angina Pectoris | Mental/Personality Disorder | Myocardial Infarction | Peripheral Arterial Disease | Substance Abuse Disorder | Pregnancy
Pre-existing conditions Specify Other	Workgroup	Pre-existing conditions Other	The free-text field related to "Pre-existing conditions" specifying other text	Alphanumeric (Free-Form Entry)
Vital Status	FITBIR	Vital Status	Status of participant/subject as alive or dead	Alive | Dead | Unknown
Incident Country	NTDS	Incident country	The country where the patient was found or to which the unit responded (or best approximation)	Two-digit numeric FIPS country code (Free-Form Entry)

DATA ELEMENT	DEFINITION SOURCE	VARIABLE TITLE	DATA DEFINITION	PERMISSIBLE VALUES
Military deployment injury indicator	FITBIR	Military deployment injury indicator	Indicator of whether the injury occurred during deployment	No | Unknown | Yes
Injury Severity Score (ISS)	FITBIR	Injury Severity Score	Score measured by injury severity score (ISS)	Numeric (Free-Form Entry)
Inter-Facility Transfer Indicator	NTDS	Inter-Facility Transfer	Was the patient transferred to your facility from another acute care facility	Yes | No
Alcohol Screen Indicator	NTDS	Alcohol Screen	A blood alcohol concentration (BAC) test was performed on the patient within 24 hours after first hospital encounter	Yes | No
Alcohol Screen Results	NTDS	Alcohol Screen Results	First recorded blood alcohol (BAC) results within 24 hours after first hospital encounter	Numeric (Free-Form Entry)
Drug Screen Indicator	FITBIR	Drug screen indicator	Indicator of whether a drug screen was performed	No | Yes
Drug Screen Results	NTDS	Drug screen	First recorded positive drug screen results within 24 hours after first hospital encounter.	AMP (Amphetamine) | BAR (Barbiturate) | BZO (Benzodiazepines) | COC (Cocaine) | mAMP (Methamphetamine) | MDMA (Ecstasy) | MTD (Methadone) | OPI (Opioid) | OXY (Oxycodone) | PCP (Phencyclidine) | TCA (Tricyclic Antidepressant) | THC (Cannabinoid) | Other, specify |None | Not tested
Drug Screen Results Specify Other	Workgroup	Drug screen other	The free-text field related to "Drug Screen Results" specifying other text	Alphanumeric (Free-Form Entry)

Supplemental 4. Data elements that met consensus for inclusion in the NTRR with their original definition source, data definition, input restrictions and PVs.
